# Sub-Cellular Dynamic Analysis of BGC823 Cells after Treatment with the Multi-Component Drug CKI Using Raman Spectroscopy

**DOI:** 10.3390/ijms241612750

**Published:** 2023-08-13

**Authors:** Wenhao Shang, Anpei Ye, Yu-Kai Tong

**Affiliations:** 1 Key Laboratory for the Physics and Chemistry of Nanodevices, School of Electronics, Peking University, Beijing 100871, China; 2 Biomed-X Center, Academy for Advanced Interdisciplinary Studies, Peking University, Beijing 100871, China

**Keywords:** subcellular, dynamic, drug CKI, Raman spectroscopy, intracellular vesicles

## Abstract

Multi-component drugs (MCDs) can induce various cellular changes covering multiple levels, from molecular and subcellular structure to cell morphology. A “non-invasive” method for comprehensively detecting the dynamic changes of cellular fine structure and chemical components on the subcellular level is highly desirable for MCD studies. In this study, the subcellular dynamic processes of gastric cancer BGC823 cells after treatment with a multi-component drug, Compound Kushen Injection (CKI), were investigated using a homemade, high-resolution, confocal Raman spectroscopy (RS) device combined with bright-field imaging. The Raman spectra of the nucleus, cytoplasm and intracellular vesicles (0.4–1 μm) were collected simultaneously for each cell treated with CKI at different times and doses. The RS measurements showed that CKI decreased the DNA signatures, which the drug is known to inhibit. Meanwhile, the CKI-induced subcellular dynamic changes in the appearance of numerous intracellular vesicles and the deconstruction of cytoplasm components were observed and discussed. The results demonstrated that high-resolution subcellular micro-Raman spectroscopy has potential for detecting fine cellular dynamic variation induced by drugs and the screening of MCDs in cancer therapy.

## 1. Introduction

“Multi-component-therapeutics” anticancer drugs, such as Traditional Chinese Medicines (TCMs), have received increased attention in drug discovery [1]. The main advantages of TCMs are their function in improving the efficacy of cancer therapy and reducing its side effects and complications [2,3], as well as modulating immune function and improving the quality of life of cancer patients during their clinical use [4]. Compound Kushen Injection (CKI) is a TCM formula approved by the National Medical Products Administration and used for the clinical treatment of various types of cancers in China [5]. The chemical fingerprint of CKI contains at least eight different components, with the primary compounds being matrine and oxymatrine [6]. It has been shown that multiple bioactive components in CKI exert an integrated anti-tumor effect through multiple targets and their associated molecular pathways [7]. CKI has been shown to suppress cell cycle and DNA repair pathways and even reduce metabolic levels in cancer cells [8]. Studies have shown that matrine can inhibit cell proliferation and induce apoptosis in various types of cancer through different molecular pathways [8,9]. In human HepG2 cells, matrine induced autophagy in a dose-dependent manner [10]. In short, the presence of CKI’s multiple bioactive ingredients causes multi-level cellular changes such as morphology variation, DNA replication/repair inhibition, cell proliferation inhibition, autophagy, and apoptosis [1,8,11,12].

Micro-Raman spectroscopy (RS), also known as molecular fingerprint spectroscopy, is a label-free and non-invasive technique for characterizing the chemical components and contents of samples [13,14]. The application of RS in drug screening and the investigation of cell response profiles has been explored for many anticancer drugs, such as cisplatin (an alkylating and DNA-binding agent), doxorubicin, vincristine, paclitaxel, etc. [15,16,17,18]. Some studies have even investigated anticancer drugs on the subcellular level [15,19,20,21]. However, the potential of RS to be used to study multi-component drugs has not been verified because of the complex cell response induced by MCD. Meanwhile, drug-induced intracellular vesicles (0.4–1 μm) have not been investigated due to the difficulty of acquiring RS data on the subcellular level.

In this study, we used CKI to demonstrate the potential of RS for multi-component drug studies on the subcellular level, including intracellular vesicle composition. Using a custom-built Raman platform with a high NA (numerical aperture) objective (100×/1.46) and a 532 nm laser, we were able to characterize the dynamics of intracellular-vesicle-related cell activities with a spatial resolution of ~200 nm. First, we treated the gastric cancer cell line BGC-823 with CKI and 5-fluorouracil (5Fu, as a reference) at different drug concentrations and durations, respectively. Second, cytotoxicity assays and trypan blue cell counting were performed to verify the CKI effects on cell proliferation and viability inhibition; then, the equivalent cytotoxic effect concentrations of CKI and 5FU were identified. Subsequently, the nuclear Raman spectra of single cells treated with CKI or 5Fu were collected to demonstrate that CKI induced DNA replication/repair and proliferation inhibition. Finally, we detected CKI-induced intracellular vesicles and analyzed the cytoplasmic dynamic process using single-cell RS and bright-field imaging.

## 2. Results and Discussions

### 2.1. Inhibition of Cellular Proliferation and Viability

**Figure 1:**
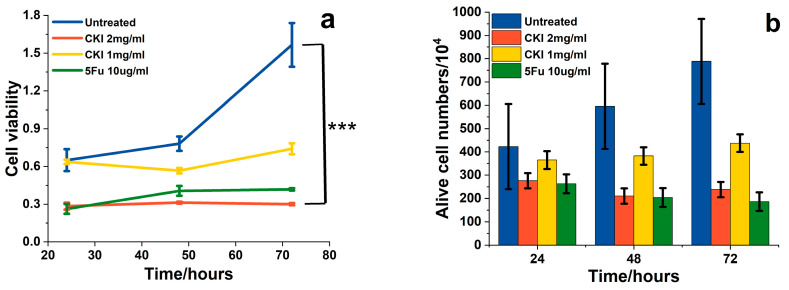
CKI inhibited proliferation and cell viability. (**a**) Inhibition of BGC823 cell viability via CKI. The viability was measured using a CCK8 kit (MTT). (**b**) Live cell counts using trypan blue staining in different drug-treated conditions. Data are represented as mean ± SD. Two-way ANOVA *** *p* < 0.01.

MTT (methyl thiazolyl tetrazolium) assays (CCK8) were performed to measure cell viability after treatment with different doses of CKI for 24, 48, and 72 h and to quantitatively validate the effect of CKI on cell proliferation in the BGC-823 gastric cancer cell line. Figure 1a shows that the cell viability of the BGC-823 cells was significantly inhibited by a high dose of CKI (2 mg/mL, based on the total alkaloid concentration in CKI) and 5Fu (10μg/mL). The cell numbers were counted via trypan blue staining (only live cells were counted). Figure 1b shows that the number of live cells was greatly decreased by CKI at 2 mg/mL and 5Fu at 10μg/mL. As shown in Figure 1a, CKI worked in a dose-dependent manner, which is consistent with the previous reports [22]. In short, the results confirmed that CKI inhibited the proliferation and viability of BGC-823 cells and showed that a CKI 2 mg/mL dose and a 5Fu 10μg/mL dose had an equivalent cytotoxic effect. However, compared to the CKI dose of 2 mg/mL and 5Fu (10μg/mL), the 1 mg/mL CKI dose clearly showed a weak inhibition of cell viability, which means that the 1 mg/mL CKI dose is too low to significantly inhibit cancer viability. Therefore, in the following spectroscopy experiments, we only used the 2 mg/mL CKI dose. 

### 2.2. The Nucleic Acid Decrease in the Cell Nucleus

**Figure 2:**
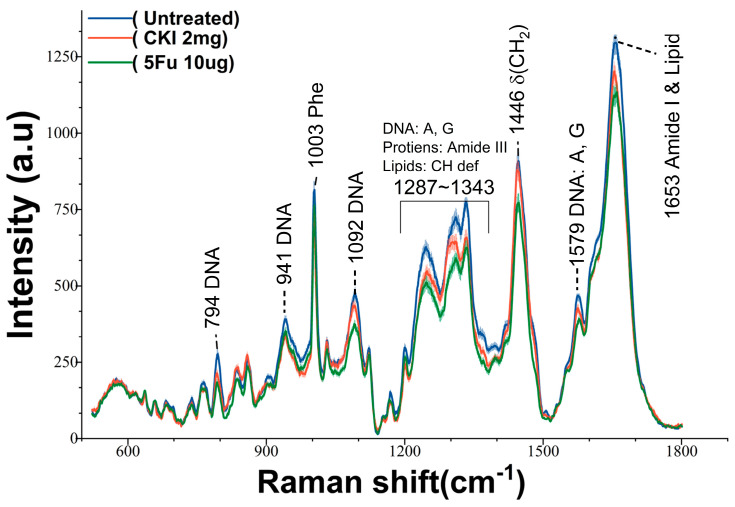
The RS of the nucleus in BGC−823 treated with CKI or 5F at 24 h (mean ± SD, the light shadow represents the SD).

The nucleus, which contains most of the cell’s DNA and is responsible for RNA (mRNA, tRNA, rRNA) synthesis, is the primary target of anticancer drugs. Previous studies have shown that 5Fu exerts its anticancer effects by inhibiting thymidylate synthase and incorporating its metabolites into RNA and DNA, thereby disrupting normal DNA and RNA processing and function, as thymidylate is required for DNA replication and repair [23]. CKI can increase the level of DNA double-strand breaks (DSBs) and inhibit DNA repair and replication [24,25,26]. We used nuclear RS to validate the drug’s effects on nucleic acid components, as shown in Figure 2. The difference in the RS between the CKI- and 5Fu-treated cells was shown by the RS peaks corresponding to nucleic acids and other cell components. In eukaryotic cells, the RS bands at 794, 941, 1092, and 1579 cm^−1^ correspond to nucleic acid components [27]. The characteristic peaks are assigned in Table S1 with reference to previous studies on different cell lines and biomolecules [27,28,29,30,31,32]. Figure 2 shows that the Raman spectral profile of the treated groups was decreased after 24 h, mainly reflected in the Raman bands assigned to nucleic acid: 794 cm^−1^ (DNA), 941 cm^−1^ (RNA), 1092 cm^−1^ (DNA), 1375 cm^−1^ (A, G, T), and 1579 cm^−1^ (DNA). The changes at 48 h, shown in Figure S1, were similar to those at 24 h. The decrease in DNA and RNA components in the nucleus confirmed the effects of CKI and 5Fu on cellular DNA and RNA.

In addition, the strong signal at 1653 cm^−1^, which includes contributions from proteins ν(C=O) (amide I) and lipids ν(C=C), decreased in intensity after the drug treatment. The decrease in proteins and lipids was also confirmed by the characteristic peaks at 1313 cm^−1^ (carbohydrates), 1334 cm^−1^ (CH_3_/CH_2_ wagging, protein), and 1446 cm^−1^ (CH_2_ bending). Surprisingly, the relative intensity of the peaks at 1455 cm^−1^ and 1653 cm^−1^ in the CKI-treated groups after 48 h tended to be the same as that in the control group. This may be due to the fact that CKI mainly affects nucleic acids rather than proteins and lipids in the nuclear region.

**Figure 3:**
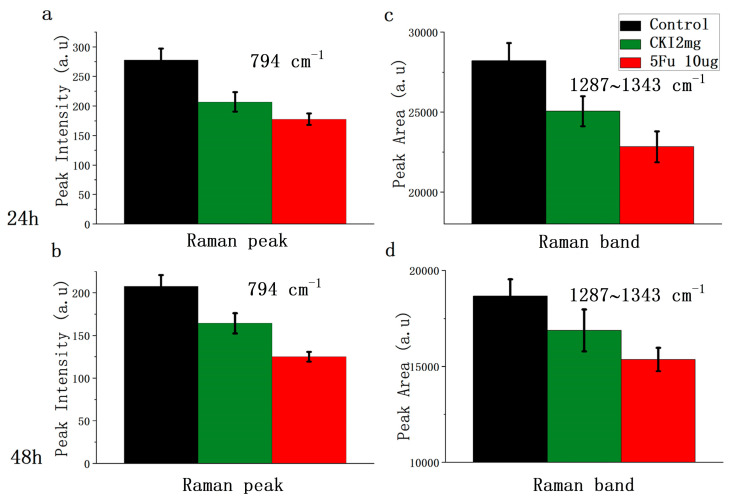
Bar plot of RS at 794 cm^−1^ and area under spectra between 1297 cm^−1^ and 1343 cm^−1^ (**a**,**b**). The RS intensity of different groups at 794 cm^−1^ (control black, CKI 2 mg green, 5Fu 10μg red) at 24 h and 48 h. (**c**,**d**). The area under spectra from 1297 cm^−1^ to 1343 cm^−1^ for different groups at 24 h and 48 h. Mean ± SD (the three groups were compared via one-way ANOVA, and *p* < 0.05 in each figure).

To quantitatively show the difference in RS signals deriving from nucleic acid between CKI and 5Fu, as an example, we compared the intensity at 794 cm^−1^ (DNA ν(OPO backbone)) and the area under the peaks at 1287–1343 cm^−1^ (mainly reflecting the A, C, G in nucleic acid), shown in Figure 3. Pairwise comparisons involving more than two groups were performed using the appropriate Bonferroni corrections, and a one-way ANOVA test was performed with *p* < 0.05. In the CKI- and 5Fu-treated cells, these nucleic acid signals were significantly reduced compared to the control group, while 5Fu had a stronger nucleic acid inhibitory effect. This is understandable, because 5Fu inhibits thymidylate synthase and incorporates its metabolites into RNA and DNA rather than being involved in DSBs and DNA repair alone. 

### 2.3. Intracellular Vesicle Accumulation

Many studies have shown that CKI can induce cell apoptosis [5,8,25], which is characterized by a number of common morphological and biochemical features, including cell shrinkage, membrane blebbing, nuclear condensation, DNA fragmentation, mitochondrial fragmentation [33]. CKI has also been shown to induce cell autophagy [10], characterized by the appearance of a double or multi-membrane cytosolic vesicle for degradation of the cellular component [33,34,35]. In addition, autophagy-induced cell death is a mode of cell survival, but sustained autophagy generally triggers apoptosis [36].

**Figure 4:**
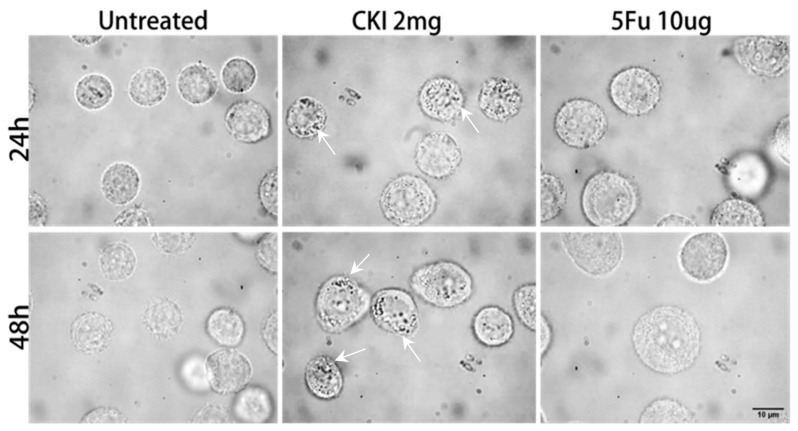
Cell morphology. Bright-field photographs of BGC823 cells in the untreated and drug-treated groups, CKI-tread group with a concentration of 2 mg/mL, and 5Fu-treated group with concentration of 10 μg/mL, respectively. Upper row represents drug treatment for 24 h and lower row represents drug treatment for 48 h.

To detect drug-induced changes in cellular morphology and intracellular vesicles (IVs) activity, we collected many cell photographs through bright-field imaging, as shown in Figure 4, and measured the corresponding RSs of the IVs and cytoplasm for the same types of cells in different treatment conditions, respectively. In the experiment, remarkable morphological changes were observed in the CKI-treated groups at both 24 h and 48. Abundant cytoplasmic vesicles (indicated by white arrows) with different sizes (0.4–1 μm) were observed only in the BGC823 cells treated with CKI. However, the size of the cells did not change significantly. As for the 5Fu-treated BGC823 cells, their size was larger than that of the untreated group, which is in agreement with our previous work [37]. Therefore, one of the keys to investigating the anticancer effect of CKI is the detection of IVs and the associated cytoplasmic dynamics. Here, considering the small size (0.4–1 μm) of the IVs, a high-spatial-resolution Raman spectrometer configured with a high NA objective (100×/1.46) with a focal spot size of 445 nm under excitation with a 532 nm laser was used to acquire the RS signals of the IVs. 

Compared with the untreated group, there was no significant change in cell size in the CKI-treated group, but this group presented many intracellular vesicles (shown as arrows), while the cell size was significantly increased in the 5Fu-treated group. This means that the mechanisms of action of CKI and 5Fu are different. 

### 2.4. RS of Intracellular Vesicles and Cytoplasm

**Figure 5:**
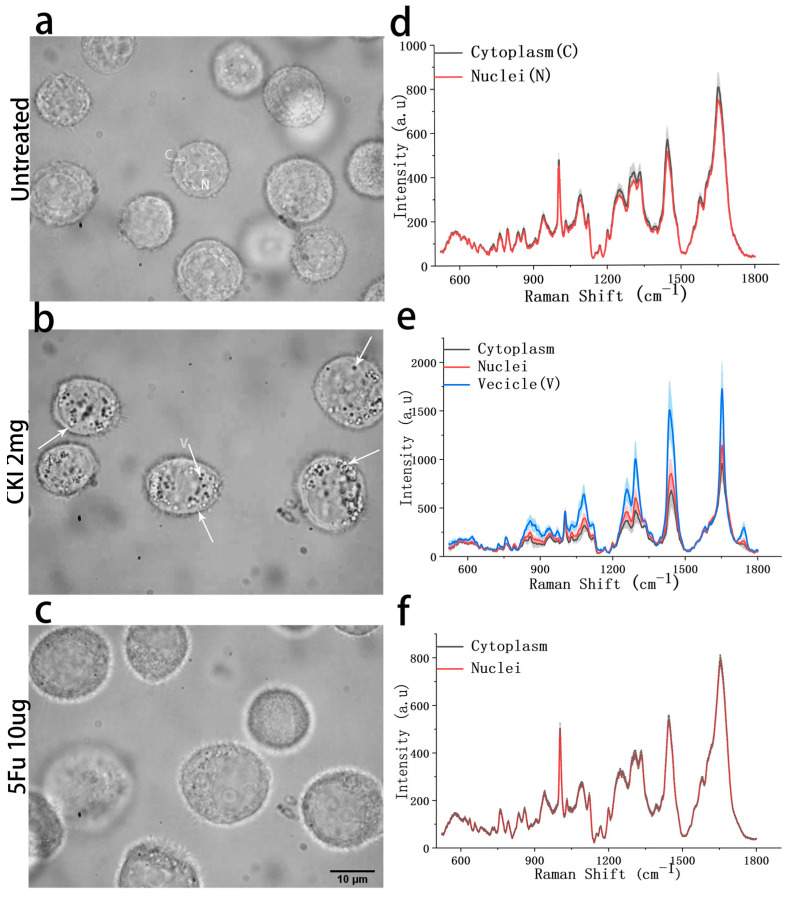
Cell images and subcellular RS spectra of BGC-823 cells. (**a**,**d**) are the images and subcellular RS spectra of cells without drug treatment. (**b**,**e**) are the images and subcellular RS spectra for CKI (2 mg/mL)-treated cells, and (**c**,**f**) are the images and subcellular RS for 5Fu (10 μg/mL)-treated cells, respectively. All data of (**b**–**f**) were collected after drug treatment for 48 h, and all RS spectra are presented with mean ± SD, where the light shadow represents the SD.

To further investigate the effect of CKI on the cells, we compared the RS results of the cytoplasm and the nucleus of the same cell. For the untreated cell group, the RS signals appeared to be same (Figure 5d). It was only in the CKI-treated group that the RS signals of the cytoplasm were significantly lower than those of the nucleus (Figure 5e). Meanwhile, RS showed that the IVs had strong signals exactly at the peaks where the cytoplasm decreased. The results at 24 h were the same as those at 48 h. This indicated that some components moved from the cytoplasm into the vesicles within the CKI-treated cells. This was consistent with the degradation of cellular components that occurs during apoptosis and autophagy.

The main peaks of the vesicles appeared at 1746 cm^−1^ (COOR), 1656 cm^−1^ (proteins), 1439 cm^−1^ (carbohydrates/lipids), 1296 cm^−1^ (lipids), 1199 cm^−1^ (proteins), 1081 cm^−1^ (proteins/lipids/glycogen), and 1030 cm^−1^ (proteins/lipids/glycogen). The signals of protein, RNA, carbohydrates, and lipids were enhanced due to the degradation of cellular components during autophagy and apoptosis. The new peaks at 1746 cm^−1^ (Figure 5e) reflected the use of a mass of phospholipids to construct vesicles. This peak is the main difference between lipids and fatty acids in cell components. It suggested that fatty acids, rather than lipids, continued to increase in the cytoplasm after CKI treatment.

**Figure 6:**
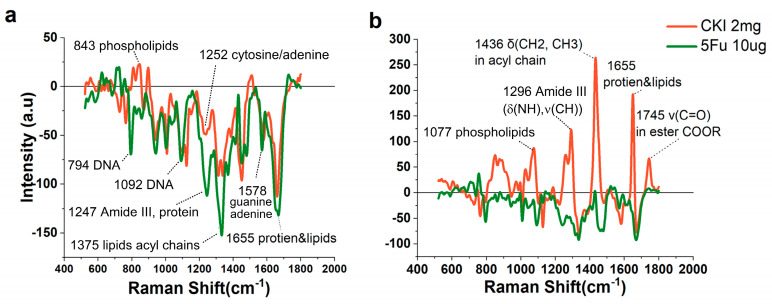
The RS of the cytoplasm of GBC−823 cells treated with CKI and 5Fu. (**a**) The RS of the cytoplasm from drug-treated cells, subtracting the RS of the cytoplasm from untreated cells at 24 h. (**b**) The RS of the cytoplasm from drug-treated cells, subtracting the RS of the cytoplasm from untreated cells at 48 h.

To clearly observe the cytoplasmic changes associated with IVs accumulation, we used the drug-treated cytoplasmic spectra minus those of the untreated group to represent the drug-mediated difference in spectral intensity. For the 5Fu-treated cells, the subtraction results are mostly negative values for both 24 h and 48 h (Figure 6a, b). The main decrease occurred at peaks of 1655 cm^−1^ (amide I, proteins) 1331 cm^−1^ (collagen), 794 cm^−1^ (DNA), 1092 cm^−1^ (DNA), 1247 cm^−1^ (amide III, protein) 1578 cm^−1^ (guanine; adenine), and 1334 cm^−1^ (protein) due to the 5Fu inhibition of thymidylate synthase and the incorporation of its metabolites into RNA and DNA, mainly targeting the S phage [23]. In contrast, for the CKI-treated (2 mg/mL) cells, the subtraction results of the cytoplasmic spectra were different for the two different time points, being mainly negative at 24 h and almost positive at 48 h. At 24 h, almost all the components were decreased, as reflected by the lower intensities at 1655 cm^−1^ (acyl chain), 1578 cm^−1^ (DNA), 1448 cm^−1^ (lipids/proteins), 1332 cm^−1^ (proteins), 1304 cm^−1^ (lipid/protein), 1252 cm^−1^ (cytosine/adenine), 1122 cm^−1^ (proteins/lipids), and 1003 cm^−1^ (Phe νs(CC)ring). Interestingly, the peak intensity at 843 cm^−1^ (phospholipids) was increased, suggesting an increase in membrane-related cell activities. This was consistent with the strong lipid signals at 48 h at the peaks of 1745 cm^−1^ (COOR), 1436 cm^−1^ (acyl chain), and 1077 cm^−1^ (typical phospholipids). This suggests that CKI may facilitate cell activities or pathways involving lipids/ fatty acids. The increase in the lipid signals and the disappearance of the RS signals around the peak of 1745 cm^−1^ mean that the increase in the lipid signals was mainly due to the increase in fatty acids in the cytoplasm. Lipids are needed to maintain cell structure, provide energy, and are involved in cell signaling. Lipid metabolism is involved in the regulation of many cellular processes, such as cell growth, proliferation, survival, apoptosis, and autophagy [38,39]. Thus, related lipid/fat acid alterations may be one of the anticancer mechanisms of CKI, given the key role of autophagy in lipid metabolism and balance in cells and the cytotoxicity of free fatty acids [40,41,42].

## 3. Materials and Methods

### 3.1. Cell Culture and Drugs

CKI, with a total alkaloid concentration of 20.4 mg/mL in 5 mL ampoules, human gastric carcinoma BGC823 cells, and 5-fluorouracil were provided by the Beijing Cancer Hospital. The cell culture method was described in our previous work [14]. Briefly, BGC-823 cells were placed in a standard culture medium, RPMI-1640 medium (Macgene, Hangzhou, China) supplemented with 10% fetal bovine serum (Tianhang Biological Technology Co., Ltd., Beijing, China), with antibiotics and cultured at 37 °C under 95% relative humidity and 5% CO_2_. After the cells reached 40% confluence, the cell culture medium was replaced with a drug-mixed medium. 

### 3.2. Cell Viability and Live Cell Counting

The cholecystokinin (CCK-8) assay experiment was performed to evaluate the anticancer effect of CKI. The wells of 96-well plates were seeded with 1 × 104 BGC-823 cells suspended in 100 μL of medium and cultured overnight. The viability of the cells treated with CKI (2 mg/mL, 1 mg/mL) and 5-FU (10 μg/mL) for 24, 48, and 72 h was then tested as previously described [14]. Meanwhile, live cell counting was performed in parallel with RS data collection using a hemocytometer since the dead cells were marked by trypan blue, but the living cells were not [43].

### 3.3. Sub-Cellular Raman Spectroscopy & Cell Bright Field Imaging 

We constructed an optical system with 532 nm laser-excited backscatter RS collection and cell bright-field imaging, as described in our previous works [44,45]. Briefly, the system was constructed from an inverted optical microscope (Axiovert 200, Zeiss, Oberkochen, Germany), a spectrometer (Specta Pro 2300i, Acton, Trenton, NJ, USA) with a liquid-nitrogen (LN)-cooled spectroscopic CCD (Spec-10, Princeton Instruments, Trenton, NJ, USA) with a spectral resolution of 4 cm^−1^, and a wavelength of 532 nm continuous-wave laser diode (Excelsior-532-200, Spectra Physics, Santa Clara, CA, USA). In particular, a high NA (numerical aperture) objective (100×/1.46 oil, N-Achroplan, Zeiss, Oberkochen, Germany) and a 100 μm pinhole were used to construct a high-resolution confocal Raman spectrometer. The size of the laser spot on the cells can be estimated as D_min_ = 1.22λ/NA, where D_min_ is the diameter of the Airy disk containing 84% of the total laser beam energy [46]. In our experiment, the wavelength of the laser was λ = 532 nm, and theoretically, the resolving power of our microscope was determined as D_min_/2 = 0.61λ/NA~222 nm. Normally, the size of BGC823 cells is 10~20 μm, and the size of the observed intracellular vesicles (IVs) is 0.4 μm to 0.9 μm. Thus, we were able to detect RS signals from the subcellular structures. It should be noted that due to the high NA objective, the system could only achieve a high spatial resolution in the x-y plane and not in the z-direction, as if the RS signals of vesicles inevitably include the contribution from the cytoplasm. However, considering the fact that the RS signal intensity is directly proportional to the excitation power, and the light intensity is greatly reduced outside the focal point of the laser when the laser is precisely focused on vesicles, and the pinhole can filter out the signal from out of the focal point. Therefore, most of the RS signals came from the IVs rather than the cytoplasm around the vesicles and were thus identified as the RS signals of the IVs. In our experiment, human BGC823 gastric cancer cells were cultured in RPMI-1640 medium (Macgene, Beijing, China) supplemented with 10% fetal bovine serum (Tianhang Biological Technology Co., Ltd., Huzhou, China) at 37 °C and 95% relative humidity with 5% CO_2_. When the cells expanded to the required size, they were digested and divided into a number of culture bottles, and certain doses of the different drugs were added to each GBC823 cell solution, respectively, and then the drug-treated cells were further incubated for a certain time. Before measuring the Raman spectra, the cell suspension was washed twice, resuspend in PBS and diluted to a certain concentration. Finally, the cell suspension was dropped on the silica coverslip for the Raman spectroscopy measurements. The laser power applied to the samples was 14 mW, and the integration time was 30 s for each spectrum’s acquisition. Meanwhile, we collected the bright-field images before the RS measurements for each cell. More than 25 cells were collected for each different condition, i.e., the untreated, CKI 2 mg/mL, and 5Fu 10 μg/mL groups. For each cell, three Raman spectra were collected from the nuclei, cytoplasm and vesicles (for the CKI-treated group).

The RS analysis was performed using OriginPro (2019b, OriginLab Corporation, Northampton, MA, USA) and in-house scripts based on R (3.6.1) and MATLAB (2019b, The MathWorks, Inc. Novi, MI, USA). For each spectrum, the cosmic rays were first removed from the raw data, and then the background envelopes were removed using 3rd-order polynomial curve fitting, followed by 5-point Savitzky–Golay smoothing, leaving a spectral region from 500 cm^−1^ to 1800 cm^−1^, which could cover the main molecular fingerprint signals. Finally, the RS was normalized to the area. As shown in our previous work [16], the area under the RS curve is a more suitable and accurate index.

## 4. Conclusions

Multi-target drugs can induce different cell responses, which are expressed in diverse cellular phenotypic changes. Here, we used high-resolution confocal micro-Raman spectroscopy combined with bright-field imaging to investigate the CKI-induced dynamic changes in BGC823 gastric cells on the single-cell level. The CKI can lead to multiple cellular changes, including DNA replication/repair inhibition, which was reflected in the reduction in nucleic-acid-related RS peaks of the cell nucleus. Meanwhile, the abundance of intracellular vesicles that appeared in the cytoplasm were observed via bright-field imaging, and high-resolution RS analysis indicates that CKI would induce cell apoptosis and autophagy. 

The RS signals of the intracellular vesicles and cytoplasm may reflect the CKI- induced degradation process of cellular components meditated by IVs. The lipid/fat acid changes showed that CKI could affect lipid metabolism. However, the detailed mechanism needs to be further investigated. Finally, this study demonstrated that high-resolution subcellular Raman spectroscopy is a powerful tool for exploring the internal complexity of cells, especially in a label-free manner to investigate multi-target drugs. 

## Data Availability

No new data were created.

## Supplementary Materials

The supporting information can be downloaded at: https://www.mdpi.com/article/10.3390/ijms241612750/s1. References [34,47,48,49,50,51,52,53] are cited in the supplementary materials.
