# Supplementary material for: Sub-Cellular Dynamic Analysis of BGC823 Cells after Treatment with the Multi-Component Drug CKI Using Raman Spectroscopy
Source: Int J Mol Sci. 2023 Aug 13;24(16):12750. doi: 10.3390/ijms241612750 (PMC10454546; doi:10.3390/ijms241612750)
Supplement: Supplementary file 1 [file ijms-24-12750-s001.zip › ijms-2498617-supplementary.pdf]

Supporting Information

Table S1. Assignments of Raman bands in spectra for cells [1-8].

Raman peaks (cm^{-1})	Components			
	Nucleic acid	Proteins	Lipids	Carbohydrates
792	DNA (ν(OPO)backbone)			
780-805	C,T,U (ν(CC)ring)			
843	Phosphodiester νs(O–P–O)			
941	RNA/ribose (ν(CC)ring)			
1003		Phe (νs(CC)ring)		
1030		Phe	ν(CC),phospholipids	(δ(CH))ν(CC), ν(CO), ν(C–OH)
1077	ν(PO_2^-)			
1092	DNA ν(PO_2^-)			
1122		ν(C-N) ν(C-C)	ν(C-N) ν(C-C)	
1199	ν(PO_2^-)	ν(C-N)		
1252		ν(C-N)		
1296		amide III (δ(NH), ν(CN))		
1313			t(CH_3CH_2)	
1334		ω(CH_3/CH_2)		
1375	A,G,T (ν(CC)ring)	glycoproteins (δ(CH_3))	lipids/acyl chains (δ(CH_3))	saccharides
1446			lipid denaturation	
1436		δ(CH_2, CH_3)	δ(CH_2, CH_3) in acyl chain	
1579	Guanine; adenine			
1660		AmideI(ν(C=O))/ α-helix	ν(C=C)	
1720-1750			ν(C=O)in ester COOR	

Abbreviation：A-adenine; G-guanine; Glu- glucose; Phe- phenylalanine; δ- in-plane deformation; γ - out-of-plane deformation; ν- stretching; ρ -rocking; t- twisting; u- wagging. s- symmetric

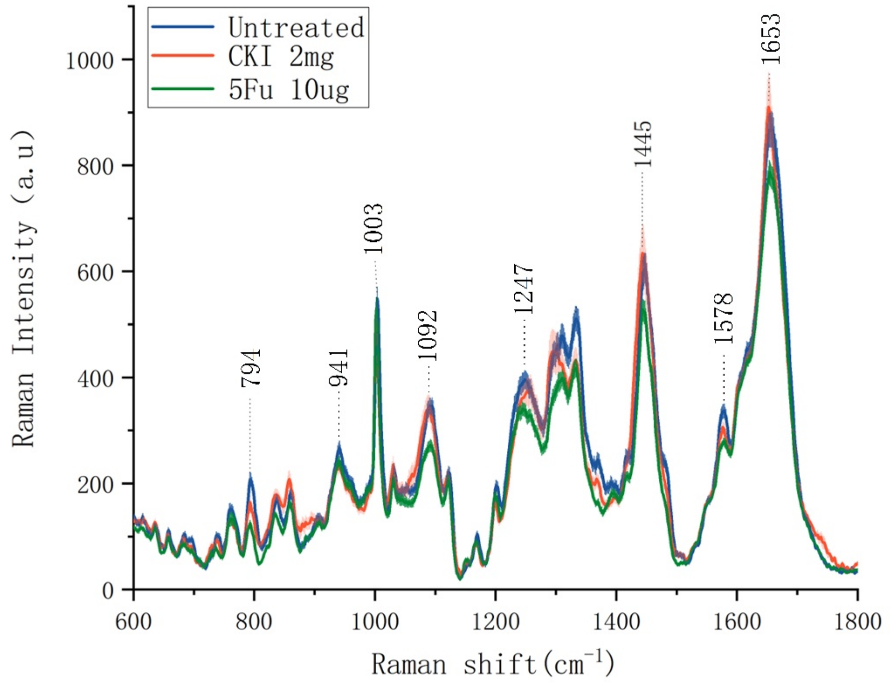

Figure S1. Raman spectrum from nucleus at 48h after drug action.

Reference

[1] S. Konorov, M. Jardon, M. Piret, et al. Raman microspectroscopy of live cells under autophagy-inducing conditions. *Analyst*, 2012, ***137***, 4662-4668.

[2] Ho C S, Jean N, Hogan C A, et al. Rapid identification of pathogenic bacteria using Raman spectroscopy and deep learning. *Nat Commun*, 2019, ***10***, 4927.

[3] Carvalho L F, Bonnier F, O'Callaghan K, et al. Raman micro-spectroscopy for rapid screening of oral squamous cell carcinoma. *Exp Mol Pathol*, 2015, ***98***, 502-9.

[4] Brauchle E, Thude S, Brucker S Y, et al. Cell death stages in single apoptotic and necrotic cells monitored by Raman microspectroscopy. *Sci Rep*, 2014, ***4***,4698.

[5] Rangan S, Kamal S, Konorov S O, et al. Types of cell death and apoptotic stages in Chinese Hamster Ovary cells distinguished by Raman spectroscopy. *Biotechnol Bioeng*, 2018, ***115***, 401-412.

[6] Eberhardt K, Beleites C, Marthandan S, et al. Raman and Infrared Spectroscopy Distinguishing Replicative Senescent from Proliferating Primary Human Fibroblast Cells by Detecting Spectral Differences Mainly Due to Biomolecular Alterations. *Analytical chemistry*, 2017, ***89,*** 2937-2947.

[7] Liendl L, Grillari J, Schosserer M. Raman fingerprints as promising markers of cellular senescence and aging. *Gero Science*, 2019, ***42***, 377-387.

[8] Choi J S, Ilin Y, Kraft M L, et al. Tracing Hematopoietic Progenitor Cell Neutrophilic Differentiation via Raman Spectroscopy. *Bioconjug Chem*, 2018, ***29***, 3121-8.
